# Plasma Sphingolipids in Acute Pancreatitis

**DOI:** 10.3390/ijms18122606

**Published:** 2017-12-04

**Authors:** Tomasz Konończuk, Bartłomiej Łukaszuk, Małgorzata Żendzian-Piotrowska, Andrzej Dąbrowski, Michalina Krzyżak, Lucyna Ostrowska, Krzysztof Kurek

**Affiliations:** 1 Department of Hygiene, Epidemiology and Ergonomics, Medical University of Bialystok, Mickiewicza 2c Street, 15-022 Bialystok, Poland; kononczuktom@onet.eu (T.K.); mzpiotrowska@gmail.com (M.Ż.-P.); michalina.krzyzak@umb.edu.pl (M.K.); 2 Department of Physiology, Medical University of Bialystok, Mickiewicza 2c Street, 15-222 Bialystok, Poland; bartlomiej.lukaszuk@umb.edu.pl; 3 Department of Gastroenterology and Internal Medicine, Medical University of Bialystok, Skłodowskiej MC 24a Street, 15-276 Bialystok, Poland; adabrows@umb.edu.pl; 4 Department of Clinical Nutrition, Medical University of Białystok, Mieszka I 4b Street, 15-054 Bialystok, Poland; lucyna@umb.edu.pl

**Keywords:** sphingolipids, acute pancreatitis, ceramide, sphingosine, sphingosine-1-phosphate

## Abstract

Acute pancreatitis (AP) is a prevalent gastrointestinal disorder associated with systemic inflammatory response syndrome and, in the case of severe AP, a mortality rate ranging from 36% to 50%. Standard clinical treatment of AP includes intensive hydration, analgesia, and management of complications. Unfortunately, the direct treatment of AP at the level of its molecular pathomechanism has not yet been established. Recent studies indicate that the sphingolipid signaling pathway may be one of the important factors contributing to the development of inflammation in pancreatic diseases. In the current study, we sought to investigate this promising route. We examined the plasma sphingolipid profile of 44 patients with acute pancreatitis, dividing them into three groups: mild, moderate and severe AP. Samples were collected from these groups at days 1, 3 and 7 following their hospital admission. We demonstrated significant changes in blood plasma sphingolipids in relation to the time course of AP. We also found an inhibition of de novo ceramide synthesis in mild and moderate AP. However, the most important and novel finding was a significant elevation in sphingosine-1-phosphate (S1P) (a downstream metabolite of ceramide) in mild AP, as well as a dramatic reduction in the lipid molecule content in the early stage (days 1 and 3) of severe AP. This strongly indicates that plasma S1P could serve as a prognostic marker of AP severity.

## 1. Introduction

Acute pancreatitis (AP) is one of the most common gastrointestinal disorders. The reported annual incidence of AP ranges from 4.9 to 35 cases per 100,000 people [1]. Pancreatic inflammation activates a cytokine cascade, which often leads to the development of systemic inflammatory response syndrome (SIRS) [2]. A prolonged duration of SIRS increases the risk of multiorgan dysfunction syndrome (MODS), a condition associated with high mortality reaching up to 39% [3,4]. To diagnose AP, at least two of the following must occur: (i) characteristic abdominal pain associated with AP; (ii) serum lipase or amylase activity at least threefold greater than the upper border of the normal limit; and (iii) the presence of typical AP lesions detected by computed tomography (CT), magnetic resonance (MR) or ultrasonography (USG) [5,6]. The 2012 Atlanta classification of acute pancreatitis established three degrees of AP disease severity. The categorization is based on the presence of transient (<48 h) organ failure, or associated local complications [7]. Thus, AP is divided into three endotypes (subtypes of a condition): The most common form is mild AP, characterized by a lack of organ failure/local complications and a very low mortality rate [8]. On the other hand, moderately severe AP is defined as the presence of transient organ failure or local complications [9]. Both mild and moderately severe AP may swiftly resolve after proper medical intervention. Finally, severe AP is characterized by a persistent (>48 h) organ failure, or the presence of local/systemic complications, and is associated with a mortality rate ranging from 36% to 50% [3].

It is clearly evident that the understanding of AP pathogenesis remains incomplete. However, recently published studies highlight that disturbances in pancreatic blood flow could induce AP onset and accelerate disease progression. In particular, the pathological features observed in the course of AP are associated with dysregulation of vascular tension, endothelial activation and injury, markedly increased vascular permeability, and increased coagulation [10]. Moreover, it has been revealed that heparin inhibits the development of ischemia/reperfusion-induced AP, and promotes pancreatic regeneration [11]. Thus, the implementation of routine hemostatic tests (e.g., platelet count, fibrinogen, fibrin degradation product (d-dimer) and clotting time) into clinical practice is reasonable and allows for a better assessment of AP prognosis [12].

Nevertheless, it was demonstrated that some pro-inflammatory cytokines, i.e., tumor necrosis factor-α (TNFα) and/or interleukins (e.g., IL-1β and IL-6), could play an essential role in AP pathogenesis, thus vitally contributing to the development of the aforementioned multiorgan dysfunction [13]. So far, the clinical treatment of AP includes intensive hydration, management of its complications and the application of analgesics [14]. Unfortunately, the direct treatment of AP molecular pathomechanisms has not yet been established. Despite the attempts to introduce anti-cytokine therapy, mortality related to acute pancreatitis remains disconcertingly high. This seems to indicate that other mediators or signaling pathways may be involved in the pathogenesis of AP. Recent studies highlight the sphingolipid signaling pathway as a potential candidate for involvement in disease pathogenesis.

Sphingolipids constitute a class of bioactive particles that are engaged in various physiological and pathological processes. They perform many distinctive biological, biophysical, and biochemical functions, participating importantly in cellular differentiation, proliferation, inflammation, and tumorigenesis [15,16,17]. The primary secondary messenger of the sphingomyelin pathway of signal transduction is ceramide, a molecule capable of stimulating phosphatases, kinases, and even some transcription factors [17]. Ceramide originates mainly from the so-called de novo synthesis pathway, or from partial sphingomyelin (SM) hydrolysis [18]. In the first stage of ceramide de novo synthesis, palmitoyl-CoA and amino acid serine combine to form 3-ketosphinganine: a lipid molecule rapidly reduced to sphinganine (SFA). SFA is acylated to dihydrocermiade, which in turn is converted into ceramide by an insertion of a trans 4,5-double bond [18]. The resultant ceramide can be further metabolized into its downstream metabolites. In the presence of the enzyme ceramidase, sphingosine (SFO) is formed. Finally, SFO may be phosphorylated to sphingosine-1-phosphate (S1P) in a reaction catalyzed by sphingosine-1-kinase (SPHK1) [19]. Despite the publishing of numerous studies concerning molecular and pathophysiological mechanisms of AP [10,20], the potential role of sphingolipid signaling pathways in AP pathophysiology remains unclear. This subject has been solely investigated in a paper by Li and co-workers [21]. The authors demonstrated that sphingosine-1-kinase expression was markedly increased in the patients with severe AP [21]. Thus, to fulfill this knowledge gap, we decided to examine whether AP affects plasma sphingolipid metabolism.

## 2. Results

### 2.1. Baseline Characteristics of the Patients (Table 1)

Between August 2015 and June 2017, 48 patients were hospitalized in the Department of Gastroenterology and Internal Medicine (Medical University of Bialystok, Bialystok, Poland) and enrolled into the study. Among them, two patients had a history of relapsing AP, one patient died before the end of the study protocol, and one declined to participate in the experiment. Finally, 44 patients were included for further examination. The mean age of the participants was 45 ± 15.6 years (range 23–78), 28 (63.6%) of them were males and 16 (36.4%) females. The main causes of AP were alcohol abuse (21 cases, 47.7%), biliary stones (17 cases, 38.6%), hypertriglyceridemia (2 cases, 4.5%), autoimmune pancreatitis (2 cases, 4.5%), groove pancreatitis (1 case, 2.3%) and azathioprine treatment (1 case, 2.3%). In accordance with the revised Atlanta classification, 18 patients (40.9%) had mild AP, 15 patients (34.1%) had moderately severe AP and 11 patients (25.0%) severe acute pancreatitis. At admission, the median white blood cells count, red blood cells count and platelets count were 12.89 ± 4.56 mm^3^, 5.08 ± 1.09 mm^3^, and 237.26 ± 156.97 mm^3^, respectively. Furthermore, at admission, the serum concentrations were as follows: median hemoglobin = 15.35 ± 3.18 g/dL, CRP = 151.63 ± 80.24 mg/L, procalcitonin = 0.46 ± 2.46 ng/mL, bilirubin 1.26 ± 4.74 mg/dL, sodium = 136.04 ± 6.92 mmol/L, potassium = 4.25 ± 1.76 mmol/L, fibrinogen = 581.16 ± 282.34 mg/dL, D-dimer = 6.12 ± 2.43 mg/L, creatinine = 1.33 ± 1.04 mg/dL and urea = 37.57 ± 34.27 mg/dL. On day 3, CRP content was significantly increased compared to day 1 (*p* < 0.05). On the other hand, on day 7, CRP concentration was markedly reduced in comparison with day 1 (*p* < 0.05). Furthermore, in comparison with day 1, gradual reductions in amylase and lipase activities on day 3 and 7 were observed (*p* < 0.05). In comparison with day 1, the concentrations of d-dimer decreased gradually between days 3 and 7 (*p* < 0.05). Finally, the activities of amylase, lipase, ALT and AST at the admission were 651.73 ± 156.23 U/L, 1427.62 ± 648.26 U/L, 117.85 ± 70.82 U/L and 149.96 ± 54.12 U/L, respectively.

### 2.2. Plasma Sphinganine Contents (pmol/mg) on the First, Third and Seventh Day after AP Onset (Figure 1)

The mean SFA concentration in the control group was 6.82 ± 1.2 pmol/mg. In comparison with the control group, the patients suffering from mild AP were characterized by a significant reduction in SFA content on day 1 (1.83 ± 0.7 pmol/mg, *p* < 0.05), day 3 (2.89 ± 0.4 pmol/mg, *p* < 0.05) and day 7 (4.52 ± 1.1 pmol/mg, *p* < 0.05). Moreover, SFA concentration in the patients with mild AP on day 7 was significantly higher than on day 1 (*p* < 0.05). In the group of patients with moderate AP, the mean SFA content on day 1 was significantly lower in comparison with the controls (3.45 ± 1.0, *p* < 0.05). On the other hand, there were no other differences between these two groups of patients between days 3 (6.78 ± 1.4 pmol/mg) and 7 (7.04 ± 0.8 pmol/mg). However, we observed a significant increase in SFA level in the moderate AP patients between the first day and seventh day of the disease (*p* < 0.05). Finally, patients with Severe AP were characterized by a significant increase in SFA content on day 1 (11.52 ± 1.7 pmol/mg, *p* < 0.05) and day 3 (10.38 ± 1.3 pmol/mg, *p* < 0.05) in comparison with the controls. No other differences between these two groups were noticed on day 7 (7.15 ± 0.6 pmol/mg). However, mean SFA concentration on the seventh day was significantly lower than on the first day of the disease (*p* < 0.05).

### 2.3. Plasma Ceramide Contents (pmol/mg) on the First, Third and Seventh Day after AP Onset (Figure 2)

The mean ceramide concentration in the control group was 7610.41 ± 840.7 pmol/mg. In comparison with the control group, the patients suffering from mild AP were characterized by a significant reduction in ceramide content on day 1 (3964.15 ± 540.5 pmol/mg, *p* < 0.05) and day 3 (5500.36 ± 438.3 pmol/mg, *p* < 0.05). No other differences between these two groups were found on day 7 (7273.28 ± 650.4 pmol/mg). However, in the case of the patients with mild AP, mean ceramide concentration on day 7 was significantly higher than on day 1 (*p* < 0.05). In the group of the patients with moderate AP, mean ceramide content was significantly lower on day 1 (4503.62 ± 338.4 pmol/mg, *p* < 0.05) and day 3 (4800.29 ± 320.4 pmol/mg, *p* < 0.05) in comparison with the control patients. On the other hand, we noticed no differences between these two groups of patients on the seventh day of the disease (6503.47 ± 650.2 pmol/mg). Moreover, we observed no significant changes in ceramide level in moderate AP patients between the first and seventh day of the disease.

Finally, the patients with severe AP were characterized by significantly increased ceramide content on day 1 (11,675.23 ± 936.2 pmol/mg, *p* < 0.05) and day 3 (9034.24 ± 650.3 pmol/mg, *p* < 0.05) in comparison with the control ones. No other differences between these two groups were noticed on day 7 (8420.13 ± 430.5 pmol/mg). However, mean ceramide concentration on the seventh day was significantly lower than on the first day of the disease (*p* < 0.05).

### 2.4. Plasma Sphingosine Contents (pmol/mg) on the First, Third and Seventh Day after AP Onset (Figure 3)

The mean SFO concentration in the control group was 53.05 ± 12.6 pmol/mg. In comparison with the control group, the patients suffering from mild AP were characterized by a significant increase in SFO content on day 1 (67.46 ± 16.4 pmol/mg, *p* < 0.05). No other differences between these two groups were observed on day 3 (56.84 ± 16.2 pmol/mg) and day 7 (50.03 ± 11.6 pmol/mg); however, in the patients with mild AP, mean SFO concentration on day 7 was significantly lower than on day 1 (*p* < 0.05).

In the group of patients with moderate AP, as compared with the control patients, mean SFO content was significantly higher on day 1 (65.25 ± 29.4 pmol/mg, *p* < 0.05). However, there were no other differences between these two groups of patients on the third (58.81 ± 19.2) and seventh day of the disease (59.12 ± 18.3 pmol/mg). Moreover, we observed no differences in SFO level in moderate AP patients in comparison between the first and seventh day of the disease.

Finally, the patients with severe AP were characterized by a significant reduction in SFO content on day 1 (18.42 ± 7.2 pmol/mg, *p* < 0.05), day 3 (25.51 ± 10.4 pmol/mg, *p* < 0.05) and day 7 (36.05 ± 12.4 pmol/mg, *p* < 0.05) in comparison with controls. Moreover, mean SFO concentration on day 7 was significantly higher than on the first day of the disease (*p* < 0.05).

### 2.5. Plasma Sphingosine-1-Phosphate Contents (pmol/mg) on the First, Third and Seventh Day after AP Onset (Figure 4)

The mean S1P concentration in the control group was 304.08 ± 56.2 pmol/mg. In comparison with the control group, the patients suffering from mild AP were characterized by a significant increase in SFO content on day 1 (402.21 ± 53.4 pmol/mg, *p* < 0.05) and day 3 (380.46 ± 72.9 pmol/mg, *p* < 0.05). No other differences between these two groups were observed on day 7 (309.57 ± 69.9 pmol/mg). However, in patients with mild AP, mean S1P concentration on day 7 was significantly higher than on day 1 (*p* < 0.05).

In the group of patients with moderate AP compared to controls, no differences in S1P levels were observed on the first (361.34 ± 46.5 pmol/mg), third (308.17 ± 64.2 pmol/mg) and seventh (316.76 ± 59.4 pmol/mg) day of the disease. Moreover, we observed no significant differences in S1P level in the moderate AP patients when comparing the seventh and first day of the disease.

Finally, the patients with severe AP were characterized by a significant reduction in S1P content on day 1 (121.86 ± 24.3 pmol/mg, *p* < 0.05) and day 3 (186.34 ± 20.4 pmol/mg, *p* < 0.05) in comparison with controls. No further differences between these two groups were observed on day 7 (310.49 ± 24.9 pmol/mg). However, in the patients with mild AP, mean S1P concentration on day 7 was significantly increased in comparison with day 1 (*p* < 0.05).

## 3. Discussion

Nowadays, acute pancreatitis is one of the most serious gastrointestinal disorders. Commonly used biomarkers for AP include serum amylase and lipase activities [22]. However, the activity of these two enzymes in isolation is insufficient for AP diagnosis, as pancreatic hyperenzymemia may also occur in numerous other pathologies [23,24]. Thus, in order to properly establish AP diagnosis, it is necessary to additionally confirm the presence of typical AP abdominal pain, or AP lesions upon radiological examination. Thus, our study only included patients that fulfilled the abovementioned criteria. Regarding AP etiology, gallstones and alcohol abuse are the two main phenomena considered to be the roots of the disease; whereas hypertriglyceridemia should be taken into account as the third plausible underlying factor. Other causes of acute pancreatitis include drug-induced, hereditary, and autoimmune AP [25]. In our study, the most common etiological factors of AP were alcohol abuse (47.7%), biliary stones (38.6%), hypertriglyceridaemia (4.5%), autoimmune pancreatitis (4.5%), groove pancreatitis (2.3%) and azathioprine treatment (2.3%). Importantly, their frequencies are consistent with those disclosed in earlier reports [1,25].

Despite the fact that previously published guidelines [25] recommend intensive hydration, pain management and prevention of complications, it seems to be obvious that the development of novel therapeutic strategies should contribute to a significant reduction of the disease mortality. Interestingly, recently published studies revealed that some drugs affecting coagulation could exert a beneficial influence on AP outcomes. Warzecha and co-workers [26] observed that treatment with low doses of vitamin K antagonist, acenocoumarol, decreased pancreatic edema, necrosis, and hemorrhage in a rodent model of rats with ischemia/reperfusion-induced AP [26]. Furthermore, the administration of the abovementioned drug significantly reduced inflammation and vacuolization of pancreatic acinar cells. These observations were accompanied by reductions in the serum lipase and amylase activities, as well as in the plasma D-dimer concentration [26]. Moreover, the treatment with acenocoumarol improved pancreatic blood flow [26]. These observations were further confirmed in a rodent model of rats with cerulean-induced AP [27]. Surprisingly, acenocoumarol seems to be effective not only in AP management, but even in the prevention of disease. This is deduced from experiments in which low-dose acenoumarol administration has been shown to reduce AP severity in rodents [28]. The patients who participated in our study were characterized by several coagulation disturbances expressed in the elevated platelet count, increased D-dimer and fibrinogen concentrations (above the upper border of the reference value). However, in accordance with the previously published guidelines [25], treatment with anticoagulants is not routinely recommended [25]. Nevertheless, observations of coagulative disturbances in the course of AP indicate that treatment with acenocoumarol or low-molecular weight heparins may demonstrate some beneficial effects.

**Figure 1:**
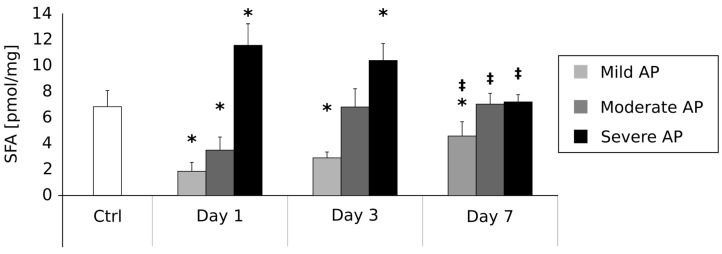
Alterations in sphinganine concentrations (pmol/mg) in the course of acute pancreatitis. * = difference vs. Control, *p* < 0.05; ^‡^ = difference vs. Day 1, *p* < 0.05.

**Figure 2:**
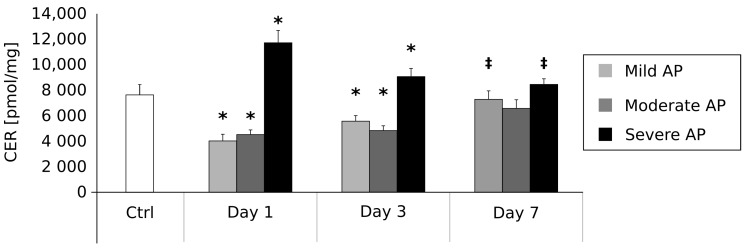
Alterations in ceramide (CER) concentrations (pmol/mg) in the course of acute pancreatitis. * = difference vs. Control, *p* < 0.05; ^‡^ = difference vs. Day 1, *p* < 0.05.

**Figure 3:**
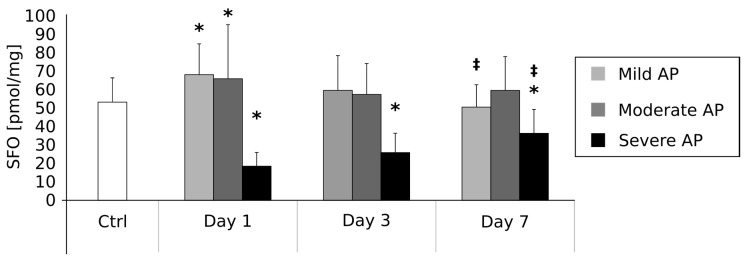
Alterations in sphingosine (SFO) concentrations (pmol/mg) in the course of acute pancreatitis. * = difference vs. Control, *p* < 0.05; ^‡^ = difference vs. Day 1, *p* < 0.05.

**Figure 4:**
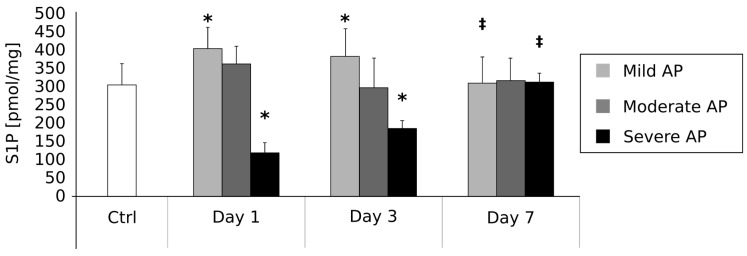
Alterations in sphingosine-1-phosphate (S1P) concentrations (pmol/mg) in the course of acute pancreatitis. * = difference vs. Control, *p* < 0.05; ^‡^ = difference vs. Day 1, *p* < 0.05.

Early prediction of AP severity is crucial to establish its proper management and, therefore, to avoid its serious complications and the resultant multiorgan dysfunction. Despite the assessment of local/systemic complications, it was recently demonstrated that Serum Soluble Fms-Like Tyrosine Kinase 1 (sFlt-1) [29], serum uromodulin [30], and serum neutrophil gelatinase-associated lipocalin (NGAL) [31] could serve as reliable early predictors of AP severity. Thus, a precise and detailed explanation of the pathophysiological and immunological processes and mechanisms of acute pancreatitis, including an investigation into different signal transduction pathways and a search for early biomarkers of the disease severity, remains a pressing issue. Hence, in the present paper, we have examined potential connections between plasma sphingolipid composition and AP. The available literature suggests that sphingolipids are involved in many pathological states of the human gastrointestinal tract [32], including inter alia, Large bowel adenomas [33], hepatic steatosis [34] or colon cancer [35]. In one of our own previously published papers, we showed that liver steatosis likely strongly depends on excessive ceramide accumulation in hepatocytes [34]. Furthermore, our 2015 study of colonic adenoma seems to confirm a vital role of sphingolipid metabolism in intestinal carcinogenesis [33]. We found a decreased ceramide and increased S1P content in the polypoid lesions characterized by a high malignancy potential. Interestingly, the inverse characteristic was found in the case of colonic polyps with a low malignancy potential [33]. Based on the above studies, it is probable that an activation of sphingolipid signaling pathways could play a pivotal role in numerous diseases related to the gastrointestinal tract. However, the existence of the postulated phenomenon in pancreatic diseases remains undetermined. In the first stage of our current research, we verified whether the hypothesized changes in sphingolipid metabolism are related to the etiology of acute pancreatitis. Surprisingly, we found no correlation between AP aetiology and change in plasma sphingolipids concentrations (Table 1). On the other hand, we observed some interesting connections between AP severity and sphingolipid metabolism (Figure 1, Figure 2, Figure 3 and Figure 4). These findings strongly suggest that the alterations in plasma sphingolipid concentrations reflect AP severity rather than its etiology. In the group of patients with mild and moderately severe AP, plasma levels of ceramide precursor sphinganine were markedly decreased in the early stage of the disease (days 1 and 3). However, after seven days, SFA content returned to the reference level (i.e., the values observed in the control individuals) in the case of the patients with moderate AP, whereas it remained markedly increased in the patients with mild AP. Furthermore, the levels of ceramide during the first three days of the disease were also decreased (mild and moderate AP). On the other hand, the patients with severe AP were characterized by significantly increased SFA and ceramide contents in the early stages (days 1 and 3) of the disease. Their levels returned to the normal values after treatment. These data strongly indicate that an inhibition of ceramide de novo synthesis takes place in the course of mild and moderate AP, but not in the case of the patients with severe AP. Indeed, patients with severe AP seemed to demonstrate an activation of ceramide de novo synthesis. Proper discussion of the observed phenomenon is virtually impossible since similar studies have not yet been performed. Nevertheless, it should be mentioned that an activation of ceramide de novo synthesis was previously demonstrated in other severe gastrointestinal diseases including pancreatic cancer [36], steatohepatitis [37] and hepatic steatosis [34].

Another novel and important finding of the current investigation is the observation of significant alterations in the blood plasma levels of ceramide downstream metabolites, i.e., sphingosine and sphingosine-1-phosphate. Previous research confirmed that both SFO and S1P act as mediators of inflammatory states, including rheumatoid arthritis [38], sepsis [39], asthma [40] and ulcerative colitis [41]. Furthermore, an inhibition of S1P synthesis via the blockage of sphingosine-1-kinase enzymatic activity decreases the inflammatory response in septic mice [39], whereas SphK1 gene deficiency acts similarly with respect to the inflammation in the case of mice with severe ulcerative colitis [42]. The above-cited papers clearly indicate a significant role of S1P in the onset and development of some inflammatory diseases. However, the possible role of ceramide downstream metabolites in AP pathogenesis is currently unexplored. The sole existing study on the topic was published by Li et al. [21]. The authors examined 22 patients with severe acute pancreatitis. The analyses encompassed the investigation of SphK1 expression (via flow cytometry), SphK enzymatic activity (via radiometric assay), and S1P concentration (via polymerase chain reaction (PCR)) in peripheral blood cells, i.e., monocytes, neutrophils and lymphocytes. Results revealed that the activity and expression of SphK1 were significantly increased in the peripheral immune cells, though only in the early stages of AP. The parameters were restored to the normal levels during the recovery period. Moreover, the authors found that the content of S1P in peripheral neutrophils and lymphocytes was markedly elevated in the early stage of severe AP. Furthermore, the abovementioned research reported a positive correlation between SphK1 expression and the severity of acute pancreatitis defined by the APACHE (Acute Physiological and Chronic Health Evaluation) II score [21]. The above presented data are consistent with our results obtained for patients with mild and moderate AP in the early stages of the disease (days 1 and 3). Ito and co-workers reported that the two main sources of S1P in the blood are platelets and erythrocytes [43]. However, the mechanisms regulating S1P release from blood cells are still poorly understood. On the other hand, in the group of patients with severe AP, we found dramatic reductions of SFO and S1P content in the early stages of the disease (days 1 and 3). Interestingly, after 7 days, SFO and S1P concentrations returned to the levels comparable with the control group, strongly suggesting that these alterations occur only in the early stages of the disease. Thus, it can be postulated that the amount of ceramide downstream metabolites (SFO and S1P) may play an important role in the diagnosis of AP in the early stages of disease. Thus, both the lipids have the potential to be used as biomarkers, which could in turn help to stratify AP patients according to risk. Interestingly, it was demonstrated that similar alterations also take place in acute diseases that are unrelated to the gastrointestinal tract. In a study by Knapp et al. [44], myocardial infarct patients were shown to be characterized by a decreased plasma S1P level. Furthermore, another study of Knapp and co-workers revealed that the aforementioned reduction in plasma sphingosine-1-phosphate concentration was accompanied by lipid accumulation in red blood cells [44]. Despite the postulated role of S1P in the pathogenesis of acute pancreatitis, some of the previously published reports indicate a possible beneficial effect of S1P with respect to the prevention of AP-related complications. Liu and co-workers [45] demonstrated that that the application of S1P or fingolimod (FTY720, a S1P analogue) greatly reduced pulmonary inflammation and injuries in the rats with sodium taurochole-induced AP [45]. The aforementioned study showed that that both S1P and FTY720 significantly reduced the activation of NF-κB (an inflammatory transcription factor) in pulmonary alveolar macrophages. Furthermore, S1P and fingolimod are capable of enhancing vascular barrier function in the lungs. This could potentially have therapeutic applications in AP, as these mechanisms may play an important role in the prevention of acute respiratory distress syndrome associated with pancreatic disease [45]. Moreover, a paper published by Muller et al. [46] demonstrated that fingolimod can decrease AP severity in rats. This positive effect of a S1P analogue may stem from the suppression of the T helper cells (Th lymphocytes), cells that normally proliferate in the early stage of the disease. The authors also revealed that FTY720 contributes to a reduction of the severity of pancreatic necrosis [46]. Moreover, recent investigation by Liu et al. [47] demonstrated that in hypertriglyceridemic apolipoprotein CIII transgenic mice, pre-treatment administration of FTY720 decreased the severity of AP [47]. However, it should be mentioned that fingolimod administration did not affect plasma content of inflammatory factors [47]. Based on the above data, one may speculate that the application of S1P and/or its analogue, fingolimod, may represent a valid novel therapeutic strategy for the prevention of AP complications. However, further studies in the area are required to resolve these issues.

In conclusion, in this study, we demonstrated significant changes in blood plasma sphingolipid concentrations accompanying the time course of acute pancreatitis. Firstly, we have found an inhibition of ceramide de novo synthesis in the case of mild and moderate AP. The most important and novel finding, however, was the observed significant elevation in S1P (a ceramide downstream metabolite) in the course of mild AP, as well as a dramatic reduction of the lipid molecule in the early stages (days 1 and 3) of severe AP. This strongly indicates that plasma S1P could serve as a reliable, cheap, and easy to apply prognostic marker of AP severity. Moreover, since S1P and its analogue fingolimod can play a protective role concerning AP complications, our findings may contribute to the development of new management strategies for this potentially fatal disease.

## 4. Materials and Methods

### 4.1. Subjects

The investigation was approved by the Ethical Committee for Human Studies of the Medical University of Bialystok. The enrolment to the study took place between August 2015 and June 2017 in the Department of Gastroenterology and Internal Medicine (Medical University of Bialystok). The inclusion criteria were (1) an individual’s age greater than 18 years old; (2) absence of any previous pancreatic disorders (based on the hospital records and an interview); (3) a confirmed diagnosis of AP. The last point was established based on the presence of at least two out of the following criteria: (i) abdominal pain typical of AP; (ii) serum lipase or amylase activity at least threefold greater than the upper border of the normal limit; and (iii) characteristic AP lesions observed in CT, MR or USG. The exclusion criteria were (1) lack of a patient’s written consent; (2) onset of symptoms more than 24 h before the admission; and (3) previous history of any pancreatic disease. The degree of AP severity was established in accordance with the revision of the Atlanta classification, and patients were grouped under three severities: mild, moderately severe, or severe [7]. All the patients received standard AP treatment, i.e., intensive intravenous hydration with lactated Ringer’s solution in the initial phase of the disease, and pain management. Endoscopic revision of biliary ducts was performed on the patients with confirmed biliary aetiology within 24 h after their admission. In the case of mild and moderately severe AP, oral feeding was started immediately after the resolution of acute symptoms (abdominal pain, nausea and vomiting). The patients with severe AP were managed with early enteral feeding. The patients with persistent multiorgan dysfunction were transferred to intensive care units. The control group consisted of healthy volunteers who were matched to the study group with respect to sex and age.

### 4.2. Serum Analyses

Blood samples were taken from a peripheral vein into the heparinized syringe on days 1, 3 and 7 after onset of AP symptoms. Samples were then centrifuged, and the procured plasma was frozen in −80 °C until further analyses. A set of commercially available kits (Abbot, IL, USA) was used to measure serum amylase, lipase, alanine transaminase (ALT) and aspartate transaminase (AST) activities, along with serum C-reactive protein, procalcitonin (PCT), bilirubin, fibrinogen, urea and creatinine contents.

### 4.3. Contents of Plasma Sphinganine (SFA), Sphinganine-1-Phosphate (S1P) and Sphingosine (SFO)

Plasma contents of SFA, S1P and SFO were measured according to the procedures described by Min and co-workers [48]. In the first step of their method, internal standards (C17-sphingosine and C17-sphingosine-1-phosphate) (Avanti Polar Lipids, Suffolk, UK) were added to the samples. Sphingoid bases were converted to their o-phthalaldehyde derivatives and subsequently examined with a HPLC system (ProStar, Varian, Inc., Cham, Switzerland) equipped with a fluorescence detector and C18 reversed-phase column (Varian, Inc., OmniSpher5, 4.6 × 150 mm).

### 4.4. Plasma Ceramide Content

A small part of the chloroform phase was moved to a fresh tube containing C17-sphingosine (Avanti Polar Lipids, Suffolk, UK, internal standard). The organic phase containing ceramide was then hydrolyzed at 90 °C for 60 min in 1 M KOH in 90% methanol solution. Sphingosine that was liberated in the abovementioned process was subsequently analyzed by HPLC. A calibration curve was prepared using *N*-palmitoylsphingosine as a standard (Avanti Polar Lipids, Suffolk, UK). The measurement of ceramide was corrected with respect to the level of free sphingosine in a given sample.

### 4.5. Statistical Analysis

The obtained results are presented in Table 1 and Figure 1, Figure 2, Figure 3 and Figure 4 as mean (bar height) and standard deviation (error bars). Any difference between groups with *p*-value less than 0.05 was considered to be statistically significant. The *p*-values were obtained after the application of ANOVA with an appropriate post-hoc test (pairwise Student’s *t*-test). If the assumptions of the above tests did not hold, a Kruskall–Wallis test with a post-hoc pairwise Mann–Whitney U test were applied. Additionally, due to multiple comparisons, a Benjamini-Hochberg correction for the obtained *p*-values was applied.

## Figures and Tables

**Table 1:** Baseline characteristic of the patients participating in the study. Number of patients, age, acute pancreatitis aetiology, disease severity, and baseline laboratory results were presented. WBC—white blood cells; HgB—hemoglobin; RBC—red blood cells; PLT—platelets; CRP—C-reactive protein; PCT—procalcitonin; ALT—alanine transaminase; AST—aspartate transaminase. * *p* < 0.05 day 7 compared with Day 1.

Variable	Mean Value		
Number of patients	*n* = 44		
Age (y)			
- mean (±SD)	45 ± 15.6		
- range, no.	23–78		
Sex, no. (%)			
- male	28 (63.6)		
- female	16 (36.4)		
Acute pancreatitis aetiology:			
- alcohol abuse	*n* = 21 (47.7%)		
- biliary	*n* = 17 (38.6%)		
- hypertriglyceridemia	*n* = 2 (4.5%)		
- autoimmune pancreatitis	*n* = 2 (4.5%)		
- groove pancreatitis	*n* = 1 (2.3%)		
- azathioprine-induced	*n* = 1 (2.3%)		
Acute pancreatitis severity:			
- mild	*n* = 18 (40.9%)		
- moderately severe	*n* = 15 (34.1%)		
- severe	*n* = 11 (25.0%)		
	Day 1	Day 3	Day 7
WBC (10^3^/mm3) (*n* = 4.0–10.0)	12.89 ± 4.56	8.02 ± 6.36	7.38 ± 5.28
HgB (g/dL) (*n* = 12.5–18.0)	15.35 ± 3.18	13.93 ± 4.37	14.21 ± 4.16
RBC (10^6^/mm^3^) (*n* = 4.2–5.4)	5.08 ± 1.09	5.26 ± 2.13	4.87 ± 2.35
PLT (10^3^/mm^3^) (*n* = 130–450)	237.26 ± 156.97	326.83 ± 138.46	190.04 ± 167.41
CRP (mg/L) (*n* ≤ 5)	151.63 ± 80.24	326.35 ± 100.93 *	40.72 ± 38.31 *
PCT (ng/ml) (*n* ≤ 0.5)	0.46 ± 2.46	0.23 ± 0.15	0.11 ± 0.73
Amylase (U/L) (*n* ≤ 90)	651.73 ± 156.23	206.44 ± 63.94 *	53.12 ± 46.63 *
Lipase (U/L) (*n* ≤ 70)	1427.62 ± 648.26	430.27 ± 158.73 *	68.32 ± 45.82 *
ALT (U/L) (*n* ≤ 50)	117.85 ± 70.82	92.34 ± 26.03	64.12 ± 30.22
AST (U/L) (*n* ≤ 50)	149.96 ± 54.12	110.03 ± 41.86	93.02 ± 23.74
Bilirubin (mg/dL) (*n* ≤ 1.1)	1.26 ± 4.74	0.78 ± 2.52	1.02 ± 0.83
Na (mmol/L) (*n* = 135–145)	136.04 ± 6.92	134.82 ± 3.15	133.54 ± 4.15
K (mmol/L) (*n* = 3.7–5.3)	4.25 ± 1.76	4.02 ± 1.82	3.98 ± 1.45
Fibrinogen (mg/dL) (*n* = 200–400)	581.16 ± 282.34	403.26 ± 173.83	390.72 ± 210.48
D-dimer (mg/L) (*n* ≤ 0.5)	6.12 ± 2.43	3.36 ± 0.14 *	1.92 ± 1.23 *
Creatinine (mg/dL) (*n* = 0.5–1.0)	1.33 ± 1.04	1.00 ± 1.45	0.8 ± 1.15
Urea (mg/dL) (*n* ≤ 50)	37.57 ± 34.27	45.18 ± 23.72	29.36 ± 27.03
